# Risk Groups for SARS-CoV-2 Infection among Healthcare Workers: Community Versus Hospital Transmission

**DOI:** 10.3390/idr13030067

**Published:** 2021-08-13

**Authors:** Fatihan Pınarlık, Zeliha Genç, Mahir Kapmaz, Süda Tekin, Önder Ergönül

**Affiliations:** 1 Department of Infectious Diseases and Clinical Microbiology, School of Medicine, Koc University, 34010 Istanbul, Turkey; fpinarlik13@ku.edu.tr (F.P.); suda.tekin@gmail.com (S.T.); 2 Graduate School of Health Sciences, Koc University, 34450 Istanbul, Turkey; 3 Koç University İşbank Center for Infectious Diseases (KUISCID), Koc University Hospital, 34010 Istanbul, Turkey; 4 Infectious Diseases and Clinical Microbiology, Koc University Hospital, 34010 Istanbul, Turkey; zakbulut@ku.edu.tr (Z.G.); mahirkapmaz@yahoo.com (M.K.)

**Keywords:** healthcare workers, COVID-19, risk factors, seroprevalence, predictor

## Abstract

Background: We aimed to detect the risk factors for SARS-CoV-2 infection among healthcare workers (HCWs) in 2020 before the vaccination era. Methods: We surveyed SARS-CoV-2 infection among the HCWs in a hospital through screening for antibody levels and the detection of viral RNA by RT-PCR between May 2020 and December 2020. Occupational and non-occupational potential predictors of disease were surveyed for the HCWs included in this study. Results: Among 1925 personnel in the hospital, 1732 were included to the study with a response rate of 90%. The overall infection rate of HCWs was 16.3% at the end of 2020, before vaccinations started. In the multivariate analysis, being janitorial staff (OR: 2.24, CI: 1.21–4.14, *p* = 0.011), being a medical secretary (OR: 4.17, CI: 2.12–8.18, *p* < 0.001), having at least one household member with a COVID-19 diagnosis (OR: 8.98, CI: 6.64–12.15, *p* < 0.001), and number of household members > 3 (OR: 1.67, CI: 1.26–2.22, *p* < 0.001) were found to be significantly associated with SARS-CoV-2 infection. Conclusions: Medical secretaries and janitorial staff were under increased risk of SARS-CoV-2 infection. The community-hospital gradient can explain the mode of transmission for infection among HCWs. In the setting of this study, community measures were less strict, whereas hospital infection control was adequate and provided necessary personal protective equipment. Increasing risk in larger households and households with diagnosed COVID-19 patient indicates the community-acquired transmission of the infection.

## 1. Introduction

The protection of HCWs from infection is strategic for the management of the pandemic. Therefore, since the beginning of the pandemic, HCWs have been screened for viral RNA and antibody levels to detect the level of infection and to determine the risk factors among HCWs. In seroprevalence studies, risk factors have been reported as being black [1,2,3,4,5,6,7], being male [5,8,9], working in frontline [9,10,11,12,13], working in an emergency department [6,7], working in an intensive care unit [14], or working in a laboratory [11]. The availability of PPE is important for protection from infection [5,15]. Some studies have reported that non-occupational risk factors such as household contact can increase seropositivity [3,6,16,17]. A recent meta-analysis revealed that male gender, ethnicity, and number of household contacts are associated with higher risks of infection [18].

Detailed and well-designed studies are needed to develop policies for the protection of HCWs. In this study, we aimed to determine the level of SARS-CoV-2 infection among HCWs and describe the predictive factors of the pre-vaccination era.

## 2. Materials and Methods

### 2.1. Study Design and Participants

We used the STROBE guideline checklist (Supplementary Materials). We intended to include all HCWs in the hospital. HCWs were reached via e-mails and internal phone calls. Participation was voluntary, and participants were free to leave the study at any time without stating an excuse. All participants were called for antibody-level testing at the end of the first (from May 2020 to the end of August 2020) and second waves (from September 2020 to the end of December 2020) of the pandemic before vaccination and the emergence of variants.

Participants with past disease proven by seropositivity or current disease spotted by RT-PCR were put in the “infection” group, whereas seronegative and PCR-negative participants were placed in the “no infection” group. All participants were surveyed for demographic, occupational, and non-occupational risk factors that might be indicators for SARS-CoV-2 infection.

We screened HCWs for past SARS-CoV-2 infection via serum antibody levels using Elecsys Anti-SARS-CoV (Roche Diagnostics) kits. For the detection of current infection in symptomatic HCWs, nasopharyngeal swap samples were tested for viral RNA via RT-PCR. Data collection was terminated by the end of 2020 after the introduction of SARS-CoV-2 vaccines in Turkey.

### 2.2. Statistical Analysis

All data were collected and stored in a secure database to protect patient confidentiality. Data were analyzed by using the Stata 16 computer program. The chi-squared test was used for binary parameters, and the Mann–Whitney U test was used for continuous variables. Statistically significant risk factors were tested with multivariate analysis, and non-significant risk factors were eliminated in a stepwise fashion.

### 2.3. Ethical Approval

The study was approved by the Republic of Turkey Ministry of Health (No: 2021-04-16T10_28_09) and the Koç University Institutional Review Board (No: 2021.254.IRB1.086).

## 3. Results

Out of 1925, 1732 (90%) HCWs responded and volunteered to participate in the study; 67.3% were female, and the median age was 28 (min: 18; max: 66). By the end of 2020, the rate of SARS-CoV-2 infection among HCWs was 16.3% before the vaccination era.

Comorbidities were reported in 4.4% of HCWs. The presence of any comorbidity was associated with infection (*p* = 0.043; Table 1). The professional distribution was dominated by nurses (48.5%) and physicians (11.7%) in the hospital. In the univariate analysis, being a medical secretary or janitorial staff were found to be associated with infection (Table 1).

For occupational risk factors, working in the pandemic ward was not found to be associated with infection. However, the inappropriate use of PPE by HCWs despite patients wearing masks was associated with an increased risk of infection. On the other hand, the proper use of PPE in HCWs performing intubation was associated with a decreased risk of infection. Additionally, having more than three years of experience in the hospital was found to be associated with a decreased risk of infection, whereas contact with a COVID-19 patient was found to be associated with an increased risk.

Three non-occupational risk factors were investigated in this study. In the univariate analysis, using public transportation was not found to be associated with infection (*p* = 0.194). However, the presence of a diagnosed COVID-19 patient in household and household size were both found to be significantly (*p* < 0.001) associated with infection.

In the multivariate analysis, being janitorial staff (OR: 2.24, CI: 1.21–4.14, *p* = 0.011), being a medical secretary (OR: 4.17, CI: 2.12–8.18, *p* < 0.001), having at least one household member with a COVID-19 diagnosis (OR: 8.98, CI: 6.64–12.15, *p* < 0.001), and number of household members > 3 (OR: 1.67, CI: 1.26–2.22, *p* < 0.001) were found to be significantly associated with SARS-CoV-2 infection (Table 2).

## 4. Discussion

In this screening study before the era of vaccinations and variants, we investigated the risk factors associated with COVID-19 infection among HCWs. We detected that being a medical secretary (OR: 4.17, CI: 2.12–8.18, *p* < 0.001) or janitorial staff (OR: 2.24, CI: 1.21–4.14, *p* = 0.011) was associated with increased risk of infection (Table 2). Though administrative staff have been found to have decreased risk in some studies [3,6,19], others have suggested that the risk of infection is higher for registrars [20], administrative employees [21], account staff [22], and janitorial staff [6,19,21,22]. The detection of being a medical secretary or janitorial staff as independent risk factors could be explained by the uncontrolled social gatherings because frontline workers are protected by strict measures. Contejean et al. suggested that close contact with coworkers without a mask can be a risk factor for seropositivity [23]. Doctors and nurses are provided with larger office spaces that prevented crowded gatherings, although medical secretaries and janitorial staff had small offices and changing rooms. Additionally, informative seminars and posters about SARS-CoV-2 infection were provided to the clinical HCWs, but critical health literacy [24] might not be achieved among medical secretaries and janitorial staff. Increased risk was not caused by the lack of PPE because all HCWs in the hospital were provided with necessary masks, shields, gowns, and other protective equipment where necessary.

In our study, among the occupational risk factors, contact with COVID-19 patients, and inappropriate use of PPE were found to be associated with an increased risk of infection. On the other hand, working experience and contact with intubated ICU patients with PPE was found to decrease the risk. These findings suggest that the transmission of SARS-CoV-2 can be blocked with the appropriate use of PPE. In settings where PPE are readily available and HCWs are trained against SARS-CoV-2 transmission, occupational transmission could be minimized. We suggest the term “community-hospital gradient” to explain the shift from the occupational-acquired form of SARS-CoV-2 to the community-acquired form. The community-hospital gradient suggests a more dynamic model, and it can be useful to explain the uncertainty that some studies [3,6,16,17] have revealed risk factors suggesting transmission from the community but whereas others [9,10,11,12,13,14] have highlighted occupational transmission. The community-hospital gradient favors hospital transmission in the setting of the strict community control of SARS-CoV-2 by mask mandates and full lockdowns, along with a lack of protective measures in the hospital caused by increased workloads and sub-optimal PPE provision. Nevertheless, the community-hospital gradient can favor community transmission for hospitals where PPE and infection control are provided, but community-level measures are less strict and the social setting promotes close contact. In such a community, household size and diagnosed patient in the household were found to be associated with an increased risk of infection. The household size factor had not been reported in previous research, although household contact was found to be associated with increased risk in several studies [3,6,16,17].

Gender and age were studied as demographic risk factors for disease. However, no statistically significant association was found. Some studies in the literature have suggested that the male gender is a risk factor for SARS-CoV-2 infection [5,8,9], though other studies [1,2,3,4,6,10,11,12,16,17,25,26,27,28,29,30] have failed to show any significant association, which matches our findings. It should be noted that HCWs belong to a working population in which the median and maximum ages are lower than those of the general population. Therefore, the low power of our study, with a small sample of older HCWs, might not have been sufficient to reach statistical significance.

Similarly, participants with comorbidities constituted only 4.4% of the studied population, thus showing that the studied HCW population is healthier than the general population. Though having at least one comorbidity was found to associated with an increased risk of SARS-CoV-2 infection in the univariate analysis, the low number of participants with chronic disease (77 participants) decreased our capacity to demonstrate any association with each comorbidity, separately. Some studies with large sample sizes were not able to show the risk associated with infection [5,9]. On the other hand, Delmas et al. suggested that diabetes is associated with higher seroprevalence (OR: 1.78, CI: 1.04–3.03) [29], whereas Goenka et al. argued that cardiovascular diseases are associated with lower seroprevalence (OR: 0.38, CI: 0.15–0.96) [26].

A strength of our study was the inclusion of 90% of the HCWs in the hospital to avoid selection bias. However, there were two main limitations of this study. The first one was the recall bias of participants. We limited recall bias by completing the surveys before serological and PCR testing, as well as by using the recording system of the occupational health unit. Another limitation was that this study only covered one hospital in Turkey, and results may have been affected by hospital- and country-specific characteristics.

## 5. Conclusions

By the end of 2020, just before the era of vaccination and variants, the rate of SARS-CoV-2 infection was 16.3% among HCWs in our hospital. Medical secretaries and janitorial staff were under an increased risk of SARS-CoV-2 infection because of their exposure in the community or because they were neglected since they were not in the frontline. Increasing risk in larger households and households with a diagnosed COVID-19 patient was found to indicate the community-acquired transmission of infection. The community-hospital gradient favors community transmission in the case of adequate protective measures being implemented in the hospital but insufficient measures being implemented in the community. Healthcare workers who are not in the frontline should also be included in education programs.

## Figures and Tables

**Table 1:** Characteristics and risk factors for SARS-CoV-2 infection.

	Total *n* = 1732 (%)	Infection *n* = 283 (%)	No Infection *n* = 1449 (%)	*p*-Value
Demographics				
Female Gender	1166 (67.3)	189 (66.8)	977 (67.4)	0.833
Median Age (IQR)		27 (24–34)	28 (24–35)	0.198
Comorbidities				
Any Comorbidity	77 (4.4)	19 (6.7)	58 (4.0)	0.043
Hypertension	20 (1.2)	3 (1.0)	17 (1.2)	0.871
Type 2 Diabetes	16 (0.9)	2 (0.7)	14 (1.0)	0.676
Renal Disease	17 (1.0)	5 (1.8)	12 (0.8)	0.143
Rheumatologica Disease	8 (0.5)	2 (0.7)	6 (0.4)	0.507
Asthma	5 (0.3)	1 (0.4)	4 (0.3)	0.825
Profession				
Nurse	840 (48.5)	123 (43.5)	717 (49.5)	0.064
Physician	203 (11.7)	24 (8.5)	179 (12.4)	0.064
Porter	129 (7.4)	23 (8.1)	106 (7.3)	0.634
Janitorial Staff	66 (3.8)	17 (6.0)	49 (3.4)	0.035
Anesthesia Technician	52 (3.0)	6 (2.1)	46 (3.2)	0.342
Laboratory Technician	52 (3.0)	11 (3.9)	41 (2.8)	0.340
Medical Secretary	46 (2.6)	20 (7.0)	26 (1.8)	<0.001
Security Staff	41 (2.4)	4 (1.4)	37 (2.6)	0.249
Radiology Technician	39 (2.2)	6 (2.1)	33 (2.3)	0.870
Pharmacy	38 (2.2)	9 (3.2)	29 (2.0)	0.216
Physiotherapist	8 (0.5)	2 (0.7)	6 (0.4)	0.507
Other	218 (12.6)	38 (13.4)	180 (12.4)	0.641
Occupational Risk Factors				
Working in COVID-19 Unit	302 (17.4)	38 (13.4)	264 (18.2)	0.052
>3 Years of Experience	882 (50.9)	123 (43.5)	759 (52.4)	0.006
Contact with Probable/Diagnosed COVID-19 Patient	1038 (59.9)	188 (66.4)	850 (58.7)	0.015
Inappropriate PPE during Invasive Procedure to the Patient	182 (10.5)	32 (11.3)	150 (10.4)	0.632
Inappropriate PPE although the Patient Has a Mask	160 (9.2)	32 (11.3)	128 (8.8)	0.009
Contact with ICU Maskless Patient with PPE	408 (23.6)	60 (21.2)	348 (24.0)	0.307
Contact with Intubated ICU Patient with PPE	293 (16.9)	35 (12.4)	258 (17.8)	0.026
Non-Occupational Risk Factors				
Diagnosed COVID-19 Patient in Household	260 (15.0)	131 (46.3)	129 (8.9)	<0.001
Household Size >3 People	792 (45.7)	158 (55.8)	634 (43.8)	<0.001
Public Transportation Use	1383 (79.8)	234 (82.7)	1149 (79.3)	0.194

**Table 2:** The predictors of SARS-CoV-2 infection among HCWs.

	Univariate Analysis				Multivariate Analysis		
Risk Factors	OR	CI	*p*	OR		CI	*p*
Janitorial Staff	1.82	1.04–3.22	0.037	2.24		1.21–4.14	0.011
Medical Secretary	4.16	2.29–7.56	<0.001	4.17		2.12–8.18	<0.001
Diagnosed Patient in Household	8.82	6.56–11.85	<0.001	8.98		6.64–12.15	<0.001
Number of Household Members >3	1.62	1.26–2.10	<0.001	1.67		1.26–2.22	<0.001

## Data Availability

Not applicable.

## Supplementary Materials

The following are available online at https://www.mdpi.com/article/10.3390/idr13030067/s1; STROBE Statement—Checklist of items that should be included in reports of cross-sectional studies.
